# Effects of a 6-Week Supervised Multimodal Exercise Program on Cancer-Related Fatigue, Quality of Life and Physical Function During Active Treatment: A Randomized Controlled Trial

**DOI:** 10.3390/cancers18060947

**Published:** 2026-03-13

**Authors:** Arturo Cano-Uceda, Paloma Pareja-García, Esther Sánchez-Rodríguez, David Fraguas-Ramos, Laura Martín-Álvarez, Rebeca Asencio-Vicente, Amaya Rivero-de la Villa, María del Mar Pérez-Pérez, Berta María Obispo-Portero, Laura Morales-Ruiz, Rosalía de Dios-Álvarez, Lara Sanchez-Barroso, Luis De Sousa-De Sousa, José Luis Maté-Muñoz, Pablo García-Fernández

**Affiliations:** 1 Faculty of Nursing, Physiotherapy and Podiatry, Complutense University of Madrid, 28040 Madrid, Spain; arcano01@ucm.es (A.C.-U.); pablga25@ucm.es (P.G.-F.); 2 Faculty of Health Sciences, Alfonso X El Sabio University, 28691 Madrid, Spain; 3 Physiotherapy, Occupational Therapy and Speech Therapy Unit, Infanta Leonor University Hospital, Vallecas, 28031 Madrid, Spain; 4 Medical Oncology Service, Infanta Leonor University Hospital, Vallecas, 28031 Madrid, Spain; 5 Rehabilitation Service, Infanta Leonor University Hospital, Vallecas, 28031 Madrid, Spain; 6 InveCuid, Instituto de Investigación Sanitaria Hospital 12 de Octubre (Imas12), 28041 Madrid, Spain

**Keywords:** therapeutic exercise, cancer, quality of life, physical function, fatigue, short-duration intervention

## Abstract

**Background:** Reduced quality of life, cancer-related fatigue, and functional impairment are common during and after oncologic treatment. Although therapeutic exercise is effective, evidence on brief, supervised programs of moderate to vigorous intensity remains limited, despite their greater clinical feasibility. This study evaluated the effectiveness of a six-week multimodal Therapeutic Exercise Program (TEP) in patients with cancer. **Methods:** A randomized controlled clinical trial (NCT05816187) was conducted with 110 patients with cancer (stages I–III), assigned to either an intervention group (supervised TEP, three sessions per week for six weeks) or a control group (usual care). The program included cardiorespiratory and strength training with intensity monitored using the Rating of Perceived Exertion (RPE), as well as stretching exercises. Quality of life (QoL) was assessed using the European Organisation for Research and Treatment of Cancer Quality of Life Questionnaire Core 30 (EORTC QLQ-C30), fatigue using the Functional Assessment of Chronic Illness Therapy-Fatigue scale (FACIT-F), functional capacity using the Six Minute Walk Test (6MWT), and muscle strength using the 30 Second Sit to Stand Test (30s-STST) and handgrip dynamometry (HGT). Analyses of covariance (ANCOVA), responder analyses based on the Minimal Clinically Important Difference (MCID), and effect sizes (ηp^2^) were performed. **Results:** The intervention group showed significant and clinically meaningful improvements in fatigue (FACIT-F: +4.53; *p* < 0.001; ηp^2^ = 0.135), global QoL (+9.22; *p* = 0.006), physical function, functional capacity (+24.16 m in the 6MWT; *p* = 0.006), and muscle strength (30s-STST: +2.71 repetitions; handgrip: +3.32 kg; *p* < 0.001). A total of 63.3% of participants were responders for fatigue compared with 13.3% in the control group (NNT = 2.00). Functional improvements showed moderate correlations with fatigue and global health status. Among symptoms, only insomnia demonstrated a significant reduction. **Conclusions:** A brief, supervised, multimodal TEP of moderate to vigorous intensity appears to be an effective, safe, and clinically relevant intervention to improve fatigue, QoL, and functional capacity in patients with cancer, with potential applicability in multidisciplinary oncologic care.

## Simple Summary

Reduced quality of life, cancer-related fatigue, and functional impairment are common problems during and after cancer treatment. To examine this issue, a randomized clinical trial was conducted with 110 patients with stage I–III cancer. Participants were randomly assigned either to an intervention group, which completed a six-week supervised exercise program, or to a control group that received usual care. The exercise program included cardiorespiratory training, strength exercises, and stretching, with intensity monitored through perceived exertion. Quality of life, fatigue, functional capacity, and muscle strength were assessed. The group that completed the exercise program showed significant and clinically meaningful improvements in fatigue, global quality of life, functional capacity, and muscle strength compared with the control group. Furthermore, a higher percentage of participants in the intervention group achieved improvements considered clinically important. Among symptoms, only insomnia showed a significant reduction. Conclusion: A brief, supervised therapeutic exercise program of moderate to vigorous intensity is safe and effective for improving fatigue, quality of life, and physical function in patients with cancer, and may be suitable for integration into routine oncologic care.

## 1. Introduction

Cancer is a disease characterized by genetic and epigenetic alterations that lead to uncontrolled cell growth, invasion, and metastasis [1,2]. In 2023, 18.5 million new cases and 10.4 million deaths were recorded worldwide, making cancer the second leading cause of death globally and projected to rise to 30.5 million new cases and 18.6 million deaths by 2050 [3]. Approximately 42% of cancer-related deaths in 2023 were associated with modifiable risk factors [3,4]. The most frequently diagnosed cancers were lung, breast, colorectal, prostate, and stomach cancer [5,6]. Breast cancer was the most commonly diagnosed cancer in women and the leading cause of cancer-related mortality in more than 100 countries, while prostate cancer was the most frequent cancer in men [5].

Cancer treatment includes surgery, chemotherapy, radiotherapy, targeted therapies, and immunotherapy, which are applied according to tumor type and stage [7,8,9,10,11]. Although these treatments are effective, they are often associated with adverse effects such as fatigue, nausea, vomiting, anemia, peripheral neuropathy, and hematologic toxicity, which negatively impact quality of life (QoL) [12,13,14,15,16]. The severity of these effects depends on the type of treatment and affects physical, emotional, and social dimensions [17,18,19,20].

QoL in patients with cancer is significantly reduced across physical, emotional, social, and functional domains, particularly among those with advanced disease, poorer general health status, or who require hospitalization [21,22]. Cancer-related fatigue is also one of the most prevalent symptoms, affecting up to 80% of patients. It is characterized by being disproportionate to activity level and by significantly interfering with daily life [23,24,25]. In addition, patients experience reductions in handgrip strength and in the distance covered during the Six Minute Walk Test (6MWT), which are associated with poorer QoL, increased risk of complications, and reduced survival [25,26,27]. Adverse effects further contribute to functional limitations, anxiety, depression, and socioeconomic difficulties, increasing the demand for health care services [25,27,28,29,30,31].

Therapeutic exercise has been established as a safe and effective intervention to improve QoL, reduce fatigue, and maintain physical function in patients with cancer [15,32,33,34,35,36,37,38,39,40,41,42,43]. Exercise programs typically combine aerobic training, resistance training, or both, with variable frequency and duration. Interventions lasting at least 12 weeks, performed three times per week and lasting 60 min or more per session, appear to produce greater benefits [15,44].

Despite the existing evidence, studies show substantial heterogeneity in the implementation of exercise programs, and there is limited information on interventions with different durations and intensities that may be more feasible and applicable during oncologic treatment. Designing short, supervised, and higher-intensity programs may maximize functional and psychological benefits while improving clinical translatability.

In this context, randomized controlled trials are needed to evaluate the effectiveness of this type of intervention. Therefore, the present study aimed to assess the effectiveness of a six-week therapeutic exercise program (TEP) combining moderate to vigorous intensity resistance training, cardiorespiratory exercise, and stretching in improving QoL, fatigue, muscle strength, and functional capacity in patients with cancer.

## 2. Methods

### 2.1. Study Design

**Figure 1:**
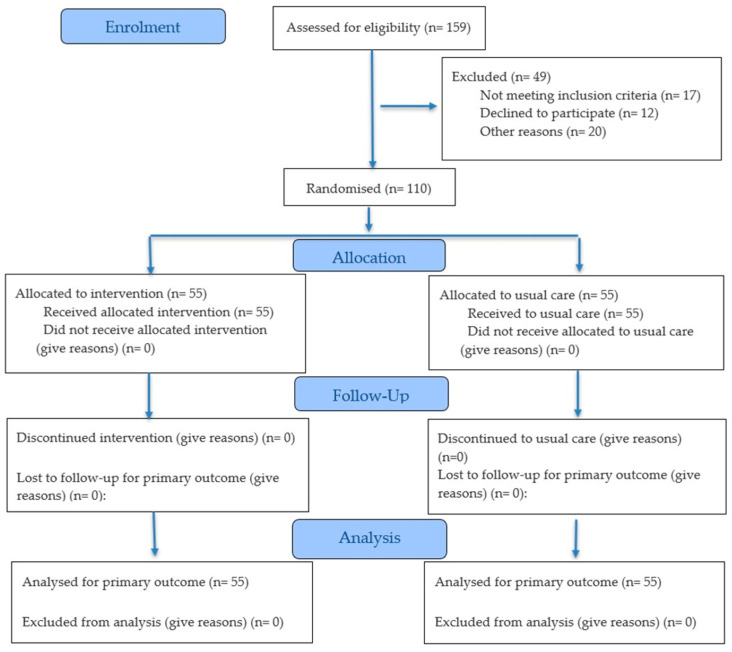
CONSORT 2025 Flow Diagram.

A randomized controlled clinical trial (NCT05816187) was conducted, including an intervention group and a control group. The study design received approval from the Ethics Committees of Hospital Universitario Infanta Leonor and Hospital Virgen de la Torre (Internal Code 012-23) and was carried out in accordance with the principles of the Declaration of Helsinki for research involving human participants [45]. The design, conduct, and reporting of the study adhered to the Consolidated Standards of Reporting Trials (CONSORT) guidelines Figure 1 [46]. Confidentiality of participants’ personal data was ensured in compliance with Organic Law 3/2018 of 5 December 2018 on the Protection of Personal Data and the guarantee of digital rights.

Patients were recruited from the hospital’s Oncology Department, where they received detailed information about the study, along with the participant information sheet and informed consent form. Those who voluntarily agreed to participate subsequently attended an initial appointment during which their questions were addressed, informed consent was signed in duplicate, and baseline measurements were obtained.

During the week prior to the start of the program, an educational session was conducted for all participants. This session explained the TEP, the planned follow-up, the frequency of assessments, and other relevant aspects. In addition, participants were provided with a dossier containing all information related to the program. Assessments were performed both in the week preceding the start of the study and in the week following completion of the intervention.

Allocation concealment, outcome assessment, and data analysis were performed blinded. Due to the nature of the intervention, blinding of participants was not feasible. A total of 110 participants were enrolled. Participants were randomly assigned (1:1) to the supervised therapeutic exercise group or the control group using a computer-generated sequence created with Microsoft Excel by an independent researcher, who maintained custody of the allocation list. Group assignment was revealed only after completion of the baseline assessment and confirmation of eligibility, preventing the recruitment team from knowing the allocation in advance. Fifty-five patients were assigned to the intervention group, which received the supervised TEP, and fifty-five to the control group, which received usual care at the health care center. Usual care included standard verbal recommendations for maintaining a healthy lifestyle, as well as a digital self-care manual containing low-intensity exercises to be performed at home. These exercise recommendations were based on the guidelines described for cancer patients by the American College of Sports Medicine [47].

The TEP lasted six weeks and consisted of three non-consecutive sessions per week. Each one-hour session included three exercise modalities: 25 min of cardiorespiratory training, 20 min of resistance training, and 15 min of stretching. In addition, participants received various self-care recommendations.

All procedures were carried out at Hospital Universitario Infanta Leonor.

### 2.2. Participants

The study included 110 men and women diagnosed with breast, prostate, and colon cancer who were receiving treatment at the Oncology Department of Hospital Universitario Infanta Leonor. All participants met the inclusion criteria, which were: (1) age between 18 and 70 years; (2) a diagnosis of stage I, II, or III cancer and having undergone, or currently undergoing, chemotherapy, radiotherapy, or hormone-based treatments within the past year; (3) no cardiopulmonary conditions that would restrict participation in physical activity; (4) no evidence of musculoskeletal disorders; and (5) an Eastern Cooperative Oncology Group (ECOG) performance status score ranging from 1 to 3. The subjects with ECOG 0 will be excluded from the study because they present an optimal functional status that is not representative of the target population for whom the intervention is intended. Their inclusion could introduce bias in the assessment of quality of life, fatigue, and functional capacity, particularly a ceiling effect that would prevent the detection of clinically meaningful improvements. In contrast, subjects with ECOG 1 and ECOG 3 represent a more appropriate functional spectrum to assess the real impact of the intervention in a population with mild to moderate functional impairment, consistent with the objectives of the study.

In addition, none of the participants met the exclusion criteria, which were defined as: (1) refusal to sign the informed consent form; (2) inability to read, comprehend, or complete the questionnaires; (3) difficulty interpreting written explanatory materials; (4) inability to follow spoken instructions (e.g., due to illiteracy, cognitive impairment such as dementia, or visual loss including blindness); (5) major neurological conditions affecting balance or motor coordination, including the presence of ataxia; (6) habitual participation in moderate-intensity physical activity exceeding 120 min per week and/or previous exposure to resistance-training programs; (7) symptomatic anemia; (8) fecal incontinence; (9) the presence of a gastrointestinal stoma; (10) decompensated cardiac disease; (11) heart failure; (12) cardiotoxicity associated with hemodynamic instability; (13) uncontrolled cardiac arrhythmias; and (14) uncontrolled arterial hypertension. A subset of women with breast cancer allocated to the intervention group (*n* = 30) had been previously described in an exploratory pre–post analysis without a control group. The present manuscript reports the main analysis of the full randomized trial, including the control group and the between-group comparison.

### 2.3. Tests and Measurements

Independent variables, including age, sex, height, weight, body mass index, cancer type, treatment received, and presence of comorbidities, were recorded on a baseline data collection form. Oxygen saturation (SpO_2_), systolic blood pressure (SBP), and diastolic blood pressure (DBP) were measured before and after each exercise session following standardized procedures.

QoL was assessed using the European Organization for Research and Treatment of Cancer Quality of Life Questionnaire Core 30 (EORTC QLQ-C30), a widely validated instrument for oncology populations and extensively used in clinical trials and observational studies [48]. The questionnaire consists of 30 items structured into multiple scales: five functional scales (physical, role, emotional, cognitive, and social functioning); three symptom scales (fatigue, pain, and nausea/vomiting); six single items assessing common cancer related symptoms (dyspnea, insomnia, appetite loss, constipation, diarrhea, and financial difficulties); and a global health status/overall QoL scale.

Most items are scored using a four-point Likert scale ranging from “not at all” to “very much,” except for the global health status scale, which uses a seven-point visual analog scale. In accordance with the EORTC scoring guidelines, all scales were converted using a linear transformation to a 0–100 metric. Higher values on the functional and global health scales denote superior functioning or enhanced quality of life, whereas higher values on the symptom scales correspond to increased symptom burden.

The questionnaire was self-administered by participants at baseline and post intervention, strictly following EORTC guidelines for administration, data handling, and interpretation to ensure comparability and validity of the results.

Fatigue was assessed using the Functional Assessment of Cancer Therapy Fatigue scale (FACIT-F), a cancer-specific instrument derived from the FACIT measurement system and widely validated for assessing fatigue severity and its functional impact [49]. The FACIT-F consists of 13 items evaluating fatigue intensity, interference, and functional consequences over the previous week, using a five-point Likert scale (0–4) ranging from “not at all” to “very much.” According to standard FACIT-F scoring procedures, item scores were summed after reversing items requiring recoding, with higher total scores indicating lower fatigue (better functional status). The questionnaire was self-administered at baseline and post intervention following the FACIT measurement system guidelines.

The two main variables in this study were overall health status on the EORTC QLQ C30 scale and fatigue measured using the FACIT-F questionnaire. The other variables in these tests were secondary, as were the variables obtained from functional capacity.

Functional capacity was assessed using the 6MWT, performed before and after the intervention. The test was conducted in accordance with the guidelines of the European Respiratory Society and the American Thoracic Society [50]. Prior to testing, participants rested in a seated position for at least 10 min, during which pulse rate, oxygen saturation, and blood pressure were measured. Participants were instructed to walk as far as possible for six minutes along a 30 m flat corridor marked with cones. A trained evaluator accompanied the participant, provided standardized encouragement, and informed them of the elapsed time at one-minute intervals. Participants were allowed to rest if necessary, although the stopwatch continued running. The number of laps and the total distance walked were recorded, and results were expressed in meters [51]. Oxygen saturation, SBP, and DBP were measured immediately before and after the test.

Lower limb strength was assessed using the 30 Second Sit to Stand Test (30s-STST), which records the number of times a participant can rise from and sit back down on a chair within 30 s. A standard armless chair with a seat height of 17 inches (43.2 cm), rubber-tipped legs, and positioned against a wall was used. Participants sat upright with arms crossed over the chest, feet shoulder-width apart and slightly behind the knees. Two practice repetitions were performed prior to the test. Participants were instructed to complete as many repetitions as possible within 30 s [52].

Overall muscle strength was evaluated using the Hand Grip Test (HGT), conducted in accordance with the recommendations of the European Society for Clinical and Economic Aspects of Osteoporosis, Osteoarthritis and Musculoskeletal Diseases (ESCEO) [53]. Participants were seated with their arms resting on the chair armrests and grasped a digital dynamometer (Saehan DHD-1, Saehan Corporation, Masan Free Trade Zone, Changwon, Republic of Korea) with the elbow flexed at 90°, the wrist in 0–30° dorsiflexion, and 0–15° ulnar deviation. Three maximal contractions were performed with the dominant hand, each lasting five seconds, with 30 s of rest between trials [54]. The mean value of the three repetitions was used for analysis.

### 2.4. Follow-Up and Data Completeness

**Figure 2:**
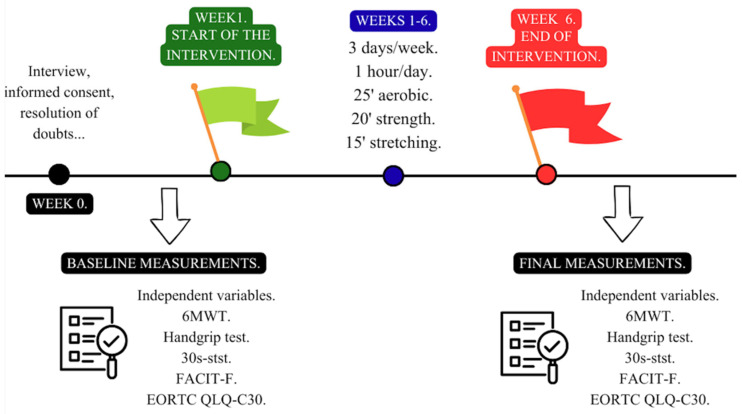
Study design. 6MWT = 6 min walking test; 30s-STST = 30 s sit to stand test; FACIT-F = Functional Assessment of Chronic Illness Therapy—Fatigue; EORTC QLQ-C30 = European Organization for Research and Treatment of Cancer Quality of Life Questionnaire Core 30.

Baseline assessments were performed during the week prior to the start of the intervention (Figure 2). Post-intervention assessments were conducted immediately after the 6-week period, within a predefined window (≤7 days) after the last supervised session in the intervention group and within the same timeframe for controls. Follow-up visits were scheduled in advance and confirmed using reminders; if a visit was missed, it was rescheduled within the assessment window. Outcome data completeness was monitored throughout the trial.

Outcome assessments were conducted at two time points only: baseline (pre-intervention) and immediately post-intervention (after 6 weeks). No outcome measurements were scheduled during the intervention period. Session attendance was recorded to quantify adherence; however, all participants were scheduled for the post-intervention assessment regardless of attendance, and no participants missed the post-intervention assessment, resulting in no missing outcome data.

Post-intervention outcomes were collected during an in-person assessment scheduled within a predefined window after the 6-week period. If a participant was temporarily unwell, the visit was postponed and rescheduled within the same window once clinically stable. If attendance had not been possible within the window, outcomes would have been recorded as missing and addressed according to the analysis plan. In this trial, no participants missed the post-intervention assessment, resulting in no missing outcome data.

### 2.5. Exercise Program

The exercise program lasted six weeks, during which participants completed three nonconsecutive 60 min sessions per week (Figure 2). Each session was structured into three components: 25 min of cardiorespiratory exercise, 20 min of resistance training, and 15 min of stretching.

#### 2.5.1. Cardiorespiratory Exercise

Aerobic training was performed using elliptical trainers, stationary bicycles, and treadmills, following a fixed three-phase structure: (1) a 5-minute warm-up, (2) a 15 min central or maintenance phase, and (3) a 5 min cool down.

During the first session, the initial workload was established as follows:

Elliptical trainer: 5 W (warm up), 25 W (maintenance), 5 W (cool down)

Stationary bicycle: 20 W (warm up), 40 W (maintenance), 20 W (cool down)

Treadmill: 3 km·h^−1^ (warm up), 5 km·h^−1^ (maintenance), 3 km·h^−1^ (cool down)

This initial workload was based on the RPE values (≈4) and HR levels (≈50–60% of HRmax) obtained from the 6MWT conducted prior to the start of the exercise program, as shown in Table 1 of the results. From the second session onward, exercise intensity was individually adjusted by modifying treadmill speed or incline, as well as resistance on the elliptical trainer and stationary bicycle, with the aim of maintaining a rating of perceived exertion between 4 and 6 on the Borg CR-10 scale. Sessions were conducted in the cardiac rehabilitation facility of Hospital Universitario Infanta Leonor. The equipment used included SCIFIT SXT7000 elliptical trainers, (SCIFIT, Tulsa, OK, USA), SCIFIT ISO1000 stationary bicycles, (SCIFIT, Tulsa, OK, USA), and Medisoft Clinical RAM 870a treadmills (Medisoft RAM Italia Srl). All devices were connected to the “CARDIAC REHABILITATION vers 2” software (Ergoline) for session monitoring.

#### 2.5.2. Resistance Training

Resistance training was performed after completion of the cardiorespiratory component and included free weights, elastic resistance bands, and body weight exercises. Two weekly sessions focused on strengthening the lower limbs and core muscles (quadriceps, hamstrings, gluteal muscles, and abdominal musculature), alternated with one session targeting the upper limbs (back, trapezius, deltoids, pectoral muscles, biceps, and triceps). Each session consisted of three sets of four exercises corresponding to the muscle groups trained.

The number of repetitions per set ranged from 10 to 15, typically progressing from 10 repetitions in the first set to 12 in the second and 15 in the third. Loads and repetitions were adapted to individual tolerance, maintaining a perceived exertion between 7 and 8 on a 10-point scale. Rest periods of 60 to 90 s were established between sets, ensuring that perceived exertion decreased to values of 4 to 5 before initiating the subsequent set.

#### 2.5.3. Stretching

Stretching exercises were performed at the end of each resistance training session. For approximately 15 min, participants carried out stretches targeting the muscle groups trained during the session. The protocol included three sets per stretch, with each position held for 30 s.

### 2.6. Sample Size

Calculated for a randomized controlled clinical trial with two parallel groups, using an analysis of covariance (ANCOVA) adjusted for the baseline value of the outcome variable. To estimate the expected effect size, evidence from a previous study evaluating the impact of an exercise program on QoL in breast cancer survivors receiving aromatase inhibitor therapy was considered [55], in which moderate to large effects were reported. To avoid overestimation of the effect, a conservative effect size corresponding to a moderate standardized between-group difference was assumed (d = 0.5).

This value was converted to an ANCOVA effect size (f) using the relationship f = d/2, resulting in f = 0.25. The calculation was performed using G*Power version 3.1, assuming a two-sided significance level of 0.05 and a statistical power of 80%, with one intervention group, one control group, and one covariate (baseline value). Based on these parameters, a minimum sample size of 108 participants was estimated to maintain the desired statistical power. To account for a potential dropout rate of 10%, a total sample size of 110 participants was planned, evenly distributed between the two groups.

### 2.7. Statistical Analyses

Statistical analyses were performed using IBM SPSS Statistics version 30.0. Participants were analyzed according to the intention-to-treat principle, whereby all participants were analyzed based on their randomized group allocation, regardless of adherence. Baseline characteristics were summarized as mean (SD) or *n* (%). If post-intervention outcome data had been missing, we planned to address missingness using multiple imputation by chained equations (including group allocation, baseline outcome values, and relevant covariates), with complete-case analyses as sensitivity checks. In the present trial, no post-intervention outcome data were missing, and, therefore, imputation was not required.

To evaluate changes in functional tests and in the EORTC QLQ-C30 and FACIT-F questionnaires after six weeks, univariate analyses of covariance (ANCOVA) were conducted, adjusting for baseline values. Normality was confirmed using the Shapiro–Wilk test, and homogeneity of variances was assessed with Levene’s test. Results are presented as mean, SD, and 95% confidence intervals (95% CI). Percentage differences between groups were calculated as ([Intervention − Control]/Control) × 100. For each comparison, the adjusted mean difference (Intervention − Control) was reported along with the 95% CI, two-sided *p* value (α = 0.05), and partial eta squared (ηp^2^) was obtained from the general linear model and interpreted as small (0.01), medium (0.06), or large (0.14) [56]. Model assumptions were verified through residual inspection and Levene’s test; when heteroscedasticity was detected, robust HC3 standard errors were applied. Associations between functional tests and questionnaire scores were examined using Pearson correlation coefficients. Effect sizes and statistical power were estimated for all analyses.

Group-level clinical relevance was defined using a margin Δ* = 0.5 × SDresidual, where SD_residual = √MS_error. Evidence of clinical superiority was considered present when the lower bound of the 95% CI for the adjusted mean difference exceeded Δ*.

In addition to the primary ANCOVA analyses, exploratory responder analyses were conducted to support clinical interpretability. Within-participant change was calculated (Δ = POST − PRE) and the individual minimal clinically important difference was defined using a distribution-based half–standard deviation criterion: MCID_ind = 0.5 × SD(Δ) [57] calculated separately for each outcome. Participants were classified as responders if they achieved an improvement ≥ MCID_ind in the expected direction (higher scores indicate improvement for EORTC QLQ-C30 Global Health Status/QoL and FACIT-F). Proportions between groups were compared using relative risk (RR), risk difference (RD), and number needed to treat (NNT = 1/RD), all with 95% CIs (with NNT CIs derived from the RD CIs). Where established anchor-based thresholds were available for key patient-reported outcomes, responder analyses were repeated as sensitivity analyses using FACIT-F improvement ≥ 4 points [58] and EORTC QLQ-C30 Global Health Status/QoL improvement ≥ 10 points [59]. Statistical significance was set at *p* < 0.05.

## 3. Results

Of the 110 randomized participants, all completed the post intervention assessment at six weeks; therefore, no losses to follow-up were recorded, and the intention-to-treat (ITT) analysis included all 110 participants in their originally assigned groups. Adherence in the intervention group was high, with 90% of participants completing at least 80% of the scheduled sessions. No intervention-related withdrawals occurred, and safety monitoring did not identify any events requiring discontinuation of the program. Among participants in the intervention group, 42 (76.4%) had breast cancer, 7 (12.7%) prostate cancer, and 6 (10.9%) colon cancer. In the control group, 44 participants had breast cancer (80%), 4 had prostate cancer (7.3%), and 7 had colon cancer (12.7%). In the breast cancer intervention group, there were 14 patients with stage I disease, 15 with stage II, and 13 with stage III. In the control group, there were 15 patients with stage I disease, 17 with stage II, and 12 with stage III. Descriptive sociodemographic data are presented in Table 1 for both the intervention and control groups. No significant between group differences were observed at baseline for age (57.6 ± 8.0 vs. 58.5 ± 7.8 years; *p* = 0.991), body weight (76.6 ± 14.2 vs. 74.5 ± 13.9 kg; *p* = 0.806), height (163.0 ± 7.1 vs. 161.0 ± 8.4 cm; *p* = 0.809), or body mass index (28.7 ± 4.8 vs. 27.6 ± 3.7 kg/m^2^; *p* = 0.797). Although the inclusion criteria indicate that patients between ECOG 1 and 3 were selected, there were no participants in either group with ECOG 3. In the control group, there were 32 participants with ECOG 1 (58.2%) and 23 with ECOG 2 (41.8%). In the intervention group, there were 35 (63.6%) and 20 (36.4%) participants, respectively.

Results of the QoL questionnaire are presented in Table 2. Using baseline values as covariates in the univariate analysis of covariance, significantly higher scores were observed in the intervention group compared with the control group for global health status (*F* = 7.845; *p* = 0.006; *η_p_*^2^ = 0.065; *SP* = 0.793), physical functioning (*F* = 64.403; *p* < 0.001; *η_p_*^2^ = 0.393; *SP* = 1.000), role functioning (*F* = 7.094; *p* = 0.009; *η_p_*^2^ = 0.059; *SP* = 0.752), and emotional functioning (*F* = 8.433; *p* = 0.004; *η_p_*^2^ = 0.069; *SP* = 0.821). For the insomnia symptom scale, scores were significantly lower in the intervention group than in the control group (*F* = 4.015; *p* = 0.048; *η_p_*^2^ = 0.035; *SP* = 0.511).

Table 3 presents the results of the FACIT-F questionnaire. Analysis of covariance confirmed significant between-group differences (*p* < 0.001) after adjusting for the baseline covariate (*F* = 18.188; *η_p_*^2^ = 0.135; *SP* = 0.988).

Table 4 presents the results of the functional capacity tests. After the exercise program, significant differences were found between the control and intervention groups for all three tests assessed: the 6MWT (F = 7.935; *p* = 0.006; ηp^2^ = 0.064; SP = 0.798), the 30s-STST (F = 22.637; *p* < 0.001; ηp^2^ = 0.162; SP = 0.997), and the HGT (F = 20.816; *p* < 0.001; ηp^2^ = 0.151; SP = 0.995), after adjustment for baseline values.

Table 5 presents the adjusted differences and responder analyses with 95% CIs for the main variables. Significant adjusted differences favoring the intervention group were observed for all variables analyzed, with statistical significance set at *p* < 0.05. For QoL, an adjusted difference of 9.22 points was observed (95% CI: 2.70–15.74; *p* = 0.006), with 47.3% responders (26/55) in the intervention group and 12.7% (7/55) in the control group (RR = 3.91, NNT = 2.89).

Regarding fatigue measured using the FACIT-F, the adjusted difference was 4.53 points (95% CI: 2.43–6.64; *p* < 0.001), with responder rates of 69.0% (38/55) in the intervention group and 14.5% (8/55) in the control group (RR = 4.75, NNT = 2.00). For strength and functional capacity outcomes, the intervention group showed an adjusted difference of 24.16 m in the 6MWT (95% CI: 7.17–41.15; *p* = 0.006) compared with the control group, with 56.3% responders (31/55) versus 23.6% (13/55), corresponding to an RR of 2.38 and an NNT of 3.33. In the 30s-STST, the adjusted difference was 2.71 repetitions (95% CI: 1.58–3.84; *p* < 0.001), with responder rates of 70.9% (39/55) in the intervention group and 27.3% (15/55) in the control group (RR = 2.60, NNT = 2.50). For HGT, the adjusted difference was 3.32 kg (95% CI: 1.88–4.76; *p* < 0.001), with 54.5% responders (30/55) in the intervention group and 12.7% (7/55) in the control group (RR = 4.29, NNT = 2.61).

Correlations were performed within the intervention group between functional tests and QoL and fatigue questionnaires. The 6MWT showed moderate correlations (0.30 ≤ |r| ≤ 0.70) with global health status on the EORTC QLQ-C30 (*r* = 0.359; *p* < 0.01) and with FACIT-F (*r* = 0.460; *p* < 0.01). Moderate correlations were also observed between HGT and global health status on the EORTC QLQ-C30 (*r* = 0.391; *p* < 0.01), as well as FACIT-F (*r* = 0.483; *p* < 0.01). In contrast, low correlations were found between the 30s-STST and FACIT-F (*r* = 0.296; *p* < 0.01) and global health status on the EORTC QLQ-C30 (*r* = 0.106; *p* < 0.01), although these associations reached statistical significance.

Responder analyses are presented in Table 5; RR, RD and NNT are reported with 95% CIs and should be interpreted cautiously given that these exploratory, sensitivity analyses were conducted as complements to the pre-specified ANCOVA to aid clinical interpretability. Sensitivity analyses using anchor-based thresholds for key patient-reported outcomes yielded conclusions consistent with the primary distribution-based responder definition.

Given that breast cancer constitutes the majority of the sample, exploratory analyses restricted to patients with breast cancer were conducted, taking into account disease stage (Table A1, Table A2 and Table A3). Table A1 describes the variables from the EORTC QLQ-C30 questionnaire, comparing the intervention and control groups after the exercise program. Significant differences were observed across all three disease stages in the primary variable global health status and in physical functioning. For two additional variables (emotional functioning and insomnia), significant differences were found only among patients with stage III disease (*p* < 0.05). However, for the other primary variable, FACIT-F, statistical significance was observed only in patients with stage I and II disease.

Regarding the functional capacity tests (6MWT and 30s-STST), significant between-group differences were found exclusively in patients with stage I disease, whereas for the HGT, such differences were limited to patients with stage II disease.

In Table A4, Table A5 and Table A6, an exploratory analysis by cancer type is presented. The results indicate that for the primary fatigue variable, as well as for functional capacity measures and for the quality-of-life questionnaire variables, role functioning, emotional functioning, and insomnia, significant differences between the control and intervention groups occurred only in patients with breast cancer. For global health status, statistical significance was observed in breast and colorectal cancer, while the differences in physical functioning were present across all three cancer types (*p* < 0.05).

## 4. Discussion

In this randomized controlled clinical trial, a six-week multimodal therapeutic exercise intervention based on aerobic exercise, resistance training, and stretching was associated with statistically significant and overall clinically relevant improvements compared with the control group. The convergent pattern of benefits observed, integrating improvements in objective functional outcomes and patient-reported measures, is consistent with the available evidence [60,61] and with oncology guidelines recommending combined aerobic and resistance exercise programs due to their impact on physical condition, functioning, QoL, and cancer-related fatigue [33,47].

The intervention group showed a significant improvement in global health status, with limited overlap of confidence intervals, which reinforces the robustness of the observed difference and suggests a clinically meaningful effect of the intervention. Nevertheless, the interindividual variability observed highlights the need to consider personal factors and to confirm these findings in studies with larger sample sizes.

Improvements in physical functioning may be related to early neuromuscular and cardiorespiratory adaptations induced by exercise, even in short duration programs, as previously reported [35,62]. Resistance training contributes to preserving or increasing muscle mass and strength, counteracting sarcopenia and functional decline associated with both cancer and its treatments [63,64,65]. Complementarily, aerobic exercise improves cardiovascular efficiency and exercise tolerance, facilitating activities of daily living and promoting greater functional autonomy [34,66,67]. The increase observed in role functioning suggests a greater ability of participants in the intervention group to perform their usual responsibilities. This finding is clinically relevant, as the maintenance of social and occupational roles represents a central component of QoL in patients with cancer. The literature indicates that this improvement may be related to reduced perceived physical limitations and increased confidence in personal capabilities, reflecting the positive impact of exercise on functional autonomy [68,69,70,71].

In our study, the improvement observed in emotional functioning may be attributed to multiple mechanisms. Physical exercise has demonstrated beneficial effects on mood through neurotransmitter regulation [72], reduction in systemic inflammation [73], and decreases in stress and anxiety [74,75]. In addition, participation in a structured program may enhance the sense of control over the disease, improve self-efficacy, and reduce kinesiophobia, factors that are particularly relevant in oncology populations [76,77].

Absence of significant changes in cognitive and social functioning, as well as in most physical symptoms, may be related to the duration of the intervention and to the limited effects of exercise on domains other than fatigue and physical function in individuals with cancer and chronic diseases [78,79,80,81,82,83]. These domains often require longer or more specific interventions and may be influenced by external factors such as family support, treatment-related limitations, or sociocultural context [84,85]. Nevertheless, a relevant reduction in insomnia was observed, suggesting that regular exercise may improve sleep quality through regulation of circadian rhythms and reductions in anxiety and daytime fatigue, with positive effects on overall well-being [86,87].

Global QoL, measured with the EORTC QLQ-C30, increased by 9.22 points, exceeding the group level margin Δ*. According to widely used interpretive criteria, this change may be considered small to moderate and clinically relevant [88]. The immediate post intervention assessment suggests that this improvement reflects the result of a cascade of preceding changes, whereby increased functional capacity, together with reduced fatigue, leads to a better overall appraisal of health status and daily life. The responder analysis (46.4% vs. 11.9%; NNT = 2.89) further supports this person-centered interpretation.

Results from the FACIT-F scale indicate that the intervention was effective in reducing cancer-related fatigue, one of the most prevalent, persistent, and limiting symptoms in oncology populations [28,89,90,91]. Fatigue reduction may be explained by physiological and functional mechanisms associated with regular exercise [24,33], such as improved cardiorespiratory efficiency and muscular oxidative capacity resulting from aerobic training, which reduces the energetic cost of daily activities. At the same time, resistance training preserves muscle mass and strength, counteracting weakness associated with inactivity, oncologic treatments, and systemic inflammation [92,93].

In addition, exercise influences fatigue through psychological and behavioral pathways, improving motivation, self-efficacy, and mood [94,95]. The improvement in sleep observed in the intervention group may act as an additional mediator [96]. This effect robustness is reflected in the marked difference in responders (63.3% vs. 13.3%; NNT = 2.00), which is consistent with the multifactorial nature of cancer-related fatigue and with previous evidence supporting supervised multicomponent exercise interventions [41,61,97].

The program also led to improvements in aerobic capacity, lower limb function, and muscle strength. The increase observed in the 6MWT is consistent with clinically meaningful changes in oncology populations and reflects a combined improvement in cardiorespiratory efficiency and reduced limitation related to muscular fatigability [98,99,100]. The responder analysis (51.7% vs. 21.7%; NNT = 3.33) suggests individually perceptible benefits in a substantial proportion of patients.

Improvements in the 30s-STST and handgrip strength are consistent with early neuromuscular adaptations induced by resistance training [101]. An increase in functional reserve implies that daily tasks require a lower percentage of maximal capacity, reducing perceived effort and promoting activity, which may indirectly contribute to reduced fatigue and improved QoL [102]. The absence of adverse changes in physiological variables indicates that these improvements occurred without an increase in physiological stress, reinforcing the safety of the intervention.

### 4.1. Limitations

This study has limitations that should be considered when interpreting the findings. First, as this was a single-center study, generalizability to other health care settings may be limited. In addition, outcomes were assessed immediately after the intervention, so the persistence of effects in the medium to long term cannot be established. Due to the nature of the intervention, participant blinding was not feasible, which may have influenced self-reported outcomes, despite the allocation concealment and blinding implemented at key stages of the process. Finally, the possibility of contamination between groups or differences in attention not exclusively attributable to supervised exercise cannot be completely ruled out. Regarding the monitoring of the exercise sessions, although the intensity of the aerobic exercise was regulated using the RPE, a more objective method would likely have involved combining it with HR measurements. Moreover, due to the different levels of attention received by the control group (digital self-management manual) and the intervention group (supervised exercise), subjective improvements between groups could arise in variables such as fatigue or quality of life as a result of patient behavior—generally unconscious—linked to the attention they receive. On the other hand, the exploratory analyses suggest that the sample studied in prostate and colorectal cancers may not be representative, and the data should be interpreted with caution. Consequently, future research should aim to include larger samples to obtain conclusive findings in these cancer types.

### 4.2. Clinical Implications

The findings suggest that a brief, supervised, multimodal program (three sessions per week for six weeks) is feasible and could be incorporated as a supportive strategy in oncologic rehabilitation for selected patients, with individualized intensity and clinical monitoring. However, its application should be considered complementary and requires confirmation in multicenter studies with greater power for subgroup analyses, assessment of implementation, adherence, required resources, and costs, as well as follow-up to determine the durability of the benefits.

## 5. Conclusions

In this randomized controlled clinical trial, a brief (six-week), supervised, multimodal TEP of moderate to vigorous intensity was associated with statistically significant and clinically relevant improvements in fatigue, global QoL, and physical function, as well as in functional capacity and muscle strength, compared with usual care. Consistently, moderate correlations were observed between improvements in functional tests and changes in fatigue and global health status, supporting convergence between objective outcomes and patient-reported measures. Overall, these findings suggest that this type of intervention may represent a useful option within supportive oncology care, particularly during active treatment. However, broader implementation should be supported by multicenter confirmation, with greater power for subgroup analyses and follow-up to establish the durability of effects and their longer-term clinical impact.

## Figures and Tables

**Table 1:** Descriptive data measurements for age and baseline physiological and anthropometric variables.

	Control (*n* = 55)	Intervention (*n* = 55)
	Mean ± SD	Mean ± SD
Age	57.6 ± 8.0	58.5 ± 7.8
Weight (kg)	76.6 ± 14.2	74.5 ± 13.9
Height (cm)	163.0 ± 7.1	161.0 ± 8.4
BMI (kg/m^2^)	28.7 ± 4.8	27.6 ± 3.7
HR rest	85.7 ± 13.8	86.4 ± 15.0
HR (bpm)	167.7 ± 5.6	167.1 ± 5.5
BPS rest	138.3 ± 19.8	135.1 ± 19.1
BPD rest	82.9 ± 12.5	81.7 ± 13.8
SpO_2_ rest	96.7 ± 3.5	96.7 ± 3.5
HR final 6MWT	105.7 ± 20.9 (63% HRmax)	109.3 ± 19.5 (65.4% HRmax)
RPE final 6MWT	5.7 ± 2.0	4.9 ± 2.3

Abbreviations: BMI = body mass index; HR = heart rate; bpm = beats per minute; BPS = blood pressure systolic; BPD = blood pressure diastolic; SpO_2_ = blood oxygen saturation; 6MWT = six-minute walk test; RPE = rate of perceived exertion; SD = standard deviation.

**Table 2:** Results for the different domains and items of the EORTC QLQ-C30 questionnaire between groups after implementation of the exercise program.

Variable		Control (M ± SD, 95% CI)	Intervention (M ± SD, 95% CI)	*p*	Control–Intervention (%)
Global Health Status	Global health status	53.39 ± 24.18 (49.59–58.69)	64.15 ± 24.24 (58.69–68.03)	0.006 *	20%
Functional Scales	Physical functioning	45.20 ± 33.56 (38.36–52.10)	85.00 ± 16.19 (77.98–91.95)	<0.001 *	88%
	Role functioning	68.93 ± 28.61 (64.58–73.69)	78.09 ± 27.00 (73.23–82.51)	0.009 *	13%
	Emotional functioning	67.80 ± 23.64 (64.77–72.29)	77.15 ± 22.31 (72.57–80.22)	0.004 *	14%
	Cognitive functioning	70.06 ± 27.30 (67.10–75.00)	75.23 ± 25.59 (70.22–78.22)	0.264	7%
	Social functioning	85.59 ± 24.06 (80.18–92.22)	83.24± 29.19 (76.49–88.74)	0.410	−3%
Symptom Scales/Items	Fatigue	42.00 ± 24.51 (36.51–45.20)	37.82 ± 28.94 (34.56–43.48)	0.560	−10%
	Nausea and vomiting	7.34 ± 12.09 (4.42–9.65)	6.66 ± 15.11 (4.30–9.67)	0.980	−9%
	Pain	48.87 ± 28.51 (42.63–52.87)	45.47 ± 30.61 (41.39–51.91)	0.767	−7%
	Dyspnea	25.42 ± 29.26 (20.28–31.09)	24.08 ± 30.50 (18.26–29.35)	0.631	−5%
	Insomnia	48.59 ± 35.73 (44.17–55.00)	42.69 ± 34.06 (36.09–47.54)	0.048 *	−12%
	Appetite loss	20.12 ± 28.57 (13.34–27.01)	13.34 ± 25.90 (6.33–20.23)	0.164	−34%
	Constipation	17.51 ± 25.03 (12.90–22.10)	15.77 ± 21.89 (11.07–20.51)	0.609	−10%
	Diarrhea	9.04 ± 18.39 (5.23–12.83)	8.47 ± 18.22 (4.58–12.38)	0.843	−6%
	Financial difficulties	25.99 ± 33.94 (24.21–27.52)	26.83 ± 32.38 (25.28–28.65)	0.358	−3%

* = significant difference between basal and post-intervention (*p* < 0.05); M = mean ± SD = standard deviation; CI = confidence intervals.

**Table 3:** Results of the FACIT-F fatigue questionnaire between the intervention and control groups after the exercise program.

Variable	Control (M ± SD, 95% CI)	Intervention (M ± SD, 95% CI)	*p*	Control–Intervention (%)
FACIT-F	31.37 ± 11.23 (30.25–33.22)	36.63 ± 11.31 (34.80–37.76)	<0.001 *	17%

FACIT-F = Functional Assessment of Chronic Illness Therapy-Fatigue; * = significant difference between groups in post-intervention (*p* < 0.05); M = mean ± SD = standard deviation; CI = confidence intervals.

**Table 4:** Analysis of functional capacity variables between the control and intervention groups after implementation of the exercise program.

Variable	Control (M ± SD, 95% CI)	Intervention (M ± SD, 95% CI)	*p*	Control–Intervention (%)
6MWT (m)	496.98 ± 88.37 (492.94–516.89)	537.01 ± 81.55 (517.89–541.05)	0.006 *	8%
RPE 6MWT	4.71 ± 2.37 (3.97–4.97)	4.03 ± 2.35 (3.78–4.77)	0.588	−14%
BPS rest 6MWT	135.38 ± 18.76 (130.90–138.12)	129.87 ± 16.36 (127.13–134.36)	0.148	−1.7%
BPD rest 6MWT	81.72 ± 12.66 (79.14–83.46)	80.32 ± 12.09 (78.57–82.89)	0.712	−1.7%
HR rest 6MWT	85.38 ± 14.63 (82.87–88.26)	86.18 ± 11.09 (83.31–88.69)	0.052	0.9%
SpO_2_ rest 6MWT	96.67 ± 3.52 (95.65–97.65)	96.48 ± 4.97 (95.50–97.50)	0.836	−0.2%
BPS final 6MWT	144.35 ± 22.75 (138.70–147.26)	141.55 ± 22.03 (138.64–147.20)	<0.001	−1.9%
BPD final 6MWT	83.72 ± 14.29 (80.50–85.49)	81.77 ± 11.89 (79.99–84.98)	0.082	−2.3%
HR final 6MWT	107.27 ± 19.06 (104.21–112.80)	105.67 ± 23.93 (100.14–108.72)	0.187	−1.5%
SpO_2_ final 6MWT	97.20 ± 2.85 (96.11–98.29)	98.22 ± 5.49 (97.13–99.31)	0.194	1.1%
30s-STST (repetitions)	13.37 ± 4.33 (12.92–14.52)	16.78 ± 4.98 (15.64–17.23)	<0.001 *	25.5%
HGT (kg)	21.42 ± 7.57 (20.37–22.41)	24.68 ± 8.65 (23.69–25.73)	<0.001 *	15.2%

6MWT = six-minute walk test; RPE= rate of perceived exertion; BPS = blood pressure systolic; BPD = blood pressure diastolic; HR: hear rate; SpO_2_ = blood oxygen saturation 30s-STST= 30 s sit-to-stand test; HGT= handgrip test; * = significant difference between basal and post-intervention (*p* < 0.05); M = mean ± SD = standard deviation; CI = confidence intervals.

**Table 5:** Adjusted between-group differences and responder analysis.

Results	EMM Control (95% CI)	EMM Intervention (95% CI)	Adjusted Difference (Intervention–Ctrl) (95% CI)	*p*-Value	ηp^2^	Group-Level Margin Δ*
6MWT (m)	504.9 (492.9–516.9)	529.1 (517.1–541.1)	24.16 (7.17–41.15)	0.006	0.064	23.36
	MCID_ind 0.5 *×* SD(Δ)	Responders-Control	Responders-Intervention	RR (95% CI)	RD (95% CI)	NNT (95% CI)
	28.0	13/55 (23.6%)	31/55 (56.3%)	2.38 (1.39–4.09)	0.300 (0.136–0.464)	3.33 (2.16–7.35)
FACIT-F	EMM Control (95% CI)	EMM Intervention (95% CI)	Adjusted difference (Intervention–Ctrl) (95% CI)	*p*-Value	ηp^2^	Group-level margin Δ*
	31.73 (30.25–33.22)	36.27 (34.78–37.75)	4.53 (2.43–6.64)	<0.001	0.135	2.91
	MCID_ind 0.5 *×* SD(Δ)	Responders-Control	Responders-Intervention	RR (95% CI)	RD (95% CI)	NNT (95% CI)
	3.21	8/55 (14.5%)	38/55 (69.0%)	4.75 (2.42–9.31)	0.500 (0.351–0.649)	2.00 (1.54–2.85)
QoL	EMM Control (95% CI)	EMM Intervention (95% CI)	Adjusted difference (Intervention–Ctrl) (95% CI)	*p*-Value	ηp^2^	Group-level margin Δ*
	54.14 (49.59–58.69)	63.36 (95% CI 58.69–68.03)	9.22 (2.70–15.74)	0.006	0.065	8.81
	MCID_ind 0.5 *×* SD(Δ)	Responders-Control	Responders-Intervention	RR (95% CI)	RD (95% CI)	NNT (95% CI)
	9.76	7/55 (12.7%)	26/55 (47.3%)	3.91 (1.85–8.29)	0.346 (0.191–0.500)	2.89 (2.00–5.23)
30s STST	EMM Control (95% CI)	EMM Intervention (95% CI)	Adjusted difference (Intervention–Ctrl) (95% CI)	*p*-Value	ηp^2^	Group-level margin Δ*
	13.72 (12.92–14.52)	16.43 (15.63–17.23)	2.71 (1.58–3.84)	<0.001	0.163	1.55
	MCID_ind 0.5 *×* SD(Δ)	Responders-Control	Responders-Intervention	RR (95% CI)	RD (95% CI)	NNT (95% CI)
	1.73	15/55 (27.3%)	39/55 (70.9%)	2.60 (1.62–4.18)	0.400 (0.237–0.563)	2.50 (1.78–4.22)
Handgrip	EMM Control (95% CI)	EMM Intervention (95% CI)	Adjusted difference (Intervention–Ctrl) (95% CI)	*p*-Value	ηp^2^	Group-level margin Δ*
	21.39 (95% CI 20.37–22.41)	24.71 (95% CI 23.69–25.73)	3.32 kg (95% CI 1.88–4.76)	<0.001	0.151	1.99
	MCID_ind 0.5 *×* SD(Δ)	Responders-Control	Responders-Intervention	RR (95% CI)	RD (95% CI)	NNT (95% CI)
	2.16	7/55 (12.7%)	30/55 (54.5%)	4.29 (2.04–8.99)	0.383 (0.233–0.534)	2.61 (1.87–4.29)

Abbreviations: 6MWT = Six-Minute Walk Test (m); FACIT-F = Functional Assessment of Chronic Illness Therapy–Fatigue (pts); QoL = Quality of Life (0–100 pts); 30 s STST = 30 s Sit-to-Stand Test (reps); Handgrip = handgrip strength (kg); EMM = estimated marginal mean (adjusted at the mean baseline); 95% CI = 95% confidence interval; ηp^2^ = partial eta squared; MCID_ind = individual minimal clinically important difference (0.5 × SD of Δ); RR = relative risk; RD = risk difference; NNT = number needed to treat; Δ = within-subject change (POST − PRE); Δ* = group-level clinical margin (=0.5 × residual SD from ANCOVA; residual SD = √MS_error); Intervention–Ctrl = intervention minus control.

## Data Availability

The original contributions presented in this study are included in the article. Further inquiries can be directed to the corresponding author.

## Appendix A

**Table A1:** Questionnaire between groups according to each stage of the disease after implementation of the exercise program in breast cancer patients.

Variable	Stage	Control (M ± SD, 95% CI)	Intervention (M ± SD, 95% CI)	*p*-Value Group	*p*-Value	ηp^2^ 1−β
Global Health Status	I	51.1 ± 22.5 (38.7–63.6)	67.3 ± 21.8 (54.7–79.9)	0.003 *	0.031 *	0.059/0.581
	II	51.5 ± 21.5 (40.4–62.5)	66.0 ± 18.8 (55.7–76.4)		0.032 *	0.058/0.578
	III	43.8 ± 22.5 (29.5–58.01)	53.8 ± 23.1 (38.3–69.3)		0.318	0.013/0.168
Functional Scales Physical functioning	I	40.4 ± 33.0 (22.2–58.7)	84.1 ± 14.0 (75.9–92.1)	<0.001 *	<0.001 *	0.226/0.997
	II	50.6 ± 31.5 (34.4–66.8)	82.8 ± 13.5 (75.3–92.3)		<0.001 *	0.140/0.941
	III	51.1 ± 30.5 (31.7–70.5)	83.6 ± 10.1 (76.9–90.4)		<0.001 *	0.152/0.958
Functional Scales Role functioning	I	67.8 ± 32.4 (49.8–85.7)	73.8 ± 28.3 (57.5–90.1)	0.035 *	0.807	0.001/0.057
	II	68.6 ± 22.0 (57.3–79.9)	77.2 ± 23.3 (64.3–90.1)		0.039 *	0.054/0.546
	III	65.3 ± 26.1 (48.7–81.8)	83.3 ± 20.1 (70.0–87.6)		0.167	0.024/0.281
Functional Scales Emotional functioning	I	65.6 ± 23.1 (52.8–78.4)	76.2 ± 13.4 (68.4–83.9)	0.008 *	0.163	0.025/0.285
	II	67.2 ± 21.5 (56.1–78.2)	71.5 ± 23.1 (58.7–84.3)		0.317	0.013/0.169
	III	56.3 ± 30.2 (37.1–75.4)	79.2 ± 32.5 (54.8–93.7)		0.029 *	0.060/0.595
Functional Scales Cognitive functioning	I	62.2 ± 35.3 (42.7–81.8)	72.6 ± 20.3 (60.9–84.3)	0.347	0.132	0.029/0.325
	II	75.5 ± 18.7 (65.9–85.1)	73.7 ± 25.8 (59.9–93.8)		0.390	0.009/0.137
	III	58.3 ± 28.0 (40.6–76.1)	76.4 ± 35.9 (49.3–93.1)		0.378	0.010/0.142
Functional Scales Social functioning	I	77.8 ± 27.9 (62.3–93.3)	88.7± 26.1 (73.6–103.7)	0.470	0.146	0.027/0.306
	II	84.3 ± 25.3 (71.3–97.3)	76.9 ± 30.6 (59.9–93.8)		0.661	0.002/0.072
	III	86.1 ± 22.3 (72.0–100.3)	81.9 ± 28.6 (60.2–97.4)		0.045 *	0.050/0.520
Symptom Scales/Items Fatigue	I	43.7 ± 23.2 (30.9–56.5)	45.6 ± 30.5 (27.9–63.2)	0.414	0.824	0.001/0.056
	II	40.5 ± 19.2 (30.6–50.4)	37.9 ± 32.5 (20.0–55.9)		0.447	0.008/0.117
	III	58.3 ± 21.3 (44.8–71.8)	34.3 ± 17.5 (22.6–46.1)		0.407	0.009/0.131
Symptom Scales/Items Nausea and vomiting	I	13.3 ± 15.7 (4.64–22.0)	10.7 ± 24.1 (−3.2–24.6)	0.733	0.857	<0.001/0.054
	II	5.9 ± 13.1 (−0.9–12.6)	10.4 ± 13.6 (2.9–17.9)		0.489	0.006/0.106
	III	6.9 ± 8.6 (1.5–12.4)	3.0 ± 6.7 (−1.5–7.6)		0.836	0.001/0.055
Symptom Scales/Items Pain	I	64.4 ± 24.3 (51.0–77.9)	53.8 ± 26.5 (38.5–69.1)	0.684	0.683	0.002/0.069
	II	48.0 ± 23.5 (36.0–60.1)	47.3 ± 30.1 (30.6–64.0)		0.208	0.021/0.241
	III	50.0 ± 32.6 (29.3–70.7)	45.5 ± 28.0 (26.7–64.3)		0.950	<0.001/0.50
Symptom Scales/Items Dyspnea	I	31.1 ± 36.7 (10.8–51.4)	32.9 ± 33.6 (13.4–52.3)	0.501	0.599	0.004/0.082
	II	27.5 ± 27.0 (13.6–41.3)	25.9 ± 33.8 (7.2–44.6)		0.0.933	<0.001/0.051
	III	36.1 ± 30.8 (17.0–55.2)	18.2 ± 22.9 (2.8–33.6)		0.506	0.006/0.101
Symptom Scales/Items Insomnia	I	60.0 ± 31.4 (42.6–77.4)	49.1 ± 35.0 (28.8–69.3)	0.010 *	0.947	<0.001/0.050
	II	41.2 ± 34.4 (23.5–58.9)	54.0 ± 34.9 (34.7–73.3)		0.932	<0.001/0.051
	III	75.0 ± 28.9 (56.7–93.3)	39.4 ± 29.1 (19.8–59.0)		<0.001 *	0.176/0.979
Symptom Scales/Items Appetite loss	I	15.6 ± 27.8 (0.2–31.0)	19.1 ± 25.2 (4.5–33.6)	0.262	0.861	<0.001/0.053
	II	15.7± 20.8 (5.0–26.4)	18.7 ± 37.2 (−1.9–39.3)		0.741	0.001/0.062
	III	36.1 ± 33.2 (15.0–57.2)	9.1 ± 21.6 (−5.4–23.6)		0.028 *	0.062/0.601
Symptom Scales/Items Constipation	I	24.4 ± 34.4 (5.4–43.5)	16.7 ± 21.7 (4.2–29.2)	0.870	0.751	0.001/0.61
	II	19.6 ± 20.6 (9.0–30.2)	18.9 ± 20.8 (7.4–30.4)		0.385	0.010/0.139
	III	16.7 ± 26.6 (−0.2–33.6)	18.2 ± 27.4 (−0.2–36.5)		0.205	0.021/0.243
Symptom Scales/Items Diarrhea	I	17.8 ± 24.18 (4.1–31.5)	11.9 ± 24.8 (−2.4–26.2)	0.903	0.879	<0.001/0.053
	II	7.8 ± 18.7 (−1.8–17.5)	9.4 ± 15.1 (1.1–17.7)		0.886	<0.001/0.052
	III	2.8 ± 9.6 (−3.3–8.9)	3.0± 10.1 (−3.7–9.8)		0.858	<0.001/0.054
Symptom Scales/Items Financial difficulties	I	37.8 ± 41.5 (14.8–60.8)	26.2 ± 35.0 (6.0–46.4)	0.566	0.985	<0.001/0.050
	II	25.5 ± 30.1 (10–41.0)	39.7 ± 33.9 (21.0–58.5)		0.892	<0.001/0.052
	III	30.6 ± 33.2 (9.5–51.7)	22.2 ± 26.0 (6.6–41.9)		0.308	0.013/0.173

* = significant difference between groups in post-intervention (*p* < 0.05); M = mean ± SD = standard deviation; CI = confidence intervals; ηp^2^ = Partial eta squared. effect size: explains what percentage of the explained variability can be specifically attributed to the effect of the treatment; 1 − *β =* statistical power: is the probability that a study will detect a real effect when that effect actually exists.

**Table A2:** Results of the FACIT-F questionnaire on fatigue among the intervention and control groups according to each stage of the disease after implementation of the exercise program in breast cancer patients.

Variable	Stage	Control (M ± SD, 95% CI)	Intervention (M ± SD, 95% CI)	*p*-Value Group	*p*-Value	ηp^2^ 1−β
FACIT-F	I	30.8 ± 11.0 (24.7–36.9)	36.8 ± 9.7 (31.2–42.4)	<0.001 *	0.008 *	0.085/0.763
	II	31.7 ± 8.8 (27.1–36.2)	33.5 ± 12.0 (26.8–40.1)		0.034 *	0.056/0.568
	III	25.1 ± 8.3 (19.8–30.3)	34.6 ± 8.6 (29.4–39.8)		0.066	0.042/0.452

FACIT-F = Functional Assessment of Chronic Illness Therapy-Fatigue; * = significant difference between groups in post-intervention (*p* < 0.05); M = mean ± SD = standard deviation; CI = confidence intervals; ηp^2^ = Partial eta squared. effect size: explains what percentage of the explained variability can be specifically attributed to the effect of the treatment; 1 − *β =* statistical power: is the probability that a study will detect a real effect when that effect actually exists.

**Table A3:** Results of functional capacity variables among the intervention and control groups according to each stage of the disease after implementation of the exercise program in breast cancer patients.

Variable	Stage	Control (M ± SD, 95% CI)	Intervention (M ± SD, 95% CI)	*p*-Value Group	*p*-Value	ηp^2^ 1−β
6MWT (meters)	I	480.4 ± 72.7 (440.1–520.7)	562.1 ± 65.3 (524.4–599.8)	0.008 *	0.031 *	0.058/0.585
	II	499.5 ± 85.6 (455.5–543.5)	523.7 ± 79.8 (479.6–568.0)		0.088	0.036/0.400
	III	498.3 ± 67.1 (455.7–541.0)	530.0 ± 78.1 (482.8–577.2)		0.383	0.010/0.140
30s-STST (repetitions)	I	13.4 ± 4.2 (11.1–15.7)	23.1 ± 27.0 (7.5–38.7)	0.042 *	0.043 *	0.051/0.531
	II	13.9 ± 4.6 (11.5–16.3)	16.9 ± 5.7 (13.7–20.0)		0.495	0.006/0.104
	III	12.6 ± 3.4 (10.4–14.7)	17.7 ± 5.4 (14.5–20.1)		0.391	0.009/0.136
HGT (kilograms)	I	17.5 ± 6.6 (13.9–21.2)	23.2 ± 5.5 (20.0–26.3)	<0.001 *	0.005	0.098/0.823
	II	19.2 ± 5.5 (16.4–22.1)	21.9 ± 8.2 (17.3–26.4)		<0.001 *	0.166/0.975
	III	19.2 ± 3.1 (17.3–21.3)	20.6 ± 5.7 (17.2–24.0)		0.567	0.004/0.088

6MWT = six-minute walk test; 30s-STST= 30 s sit-to-stand test; HGT= handgrip test; * = significant difference between groups in post-intervention (*p* < 0.05); M = mean ± SD = standard deviation; CI = confidence intervals; ηp^2^ = Partial eta squared. effect size: explains what percentage of the explained variability can be specifically attributed to the effect of the treatment; 1 − *β =* statistical power: is the probability that a study will detect a real effect when that effect actually exists.

**Table A4:** Results for the different domains and items of the EORTC QLQ-C30 questionnaire among the intervention and control groups according to each stage of the disease after implementation of the exercise program in cancer patients.

Variable	Type of Cancer	Control (M ± SD, 95% CI)	Intervention (M ± SD, 95% CI)	*p*-Value Group	*p*-Value	ηp^2^ 1−β
Global Health Status	Colorectal	56.9 ± 31.8 (23.6–90.3)	66.7 ± 23.6 (29.2–79.9)	0.191	0.037 *	0.044/0.555
	Breast	49.2 ± 21.9 (42.6–55.9)	63.1 ± 21.4 (56.3–69.9)		0.005 *	0.077/0.809
	Prostate	87.5 ± 16.0 (62.1–112.9)	65.7 ± 36.1 (32.1–98.9)		0.220	0.015/0.231
Functional Scales Physical functioning	Colorectal	35.6 ± 38.1 (−4.4–75.5)	80.0 ± 12.2 (60.6–99.4)	<0.001 *	0.011 *	0.064/0.731
	Breast	47.3 ± 31.4 (37.7–56.8)	84.7 ± 14.6 (79.5–87.5)		<0.001 *	0.301/1.000
	Prostate	28.3 ± 48.2 (−48.4–105.0)	86.7 ± 23.1 (65.3–108.0)		<0.001 *	0.109/0.932
Functional Scales Role functioning	Colorectal	58.3 ± 46.8 (9.2–107.5)	75.0 ± 21.5 (40.8–109.2)	0.207	0.357	0.009/0.150
	Breast	67.4 ± 26.4 (59.4–75.5)	77.8 ± 24.0 (69.2–83.7)		0.029 *	0.047/0.595
	Prostate	95.8 ± 8.3 (82.6–109.1)	81.0 ± 37.8 (46.0–115.9)		0.878	<0.001/0.053
Functional Scales Emotional functioning	Colorectal	77.8 ± 17.2 (59.7–95.8)	79.2 ± 16.0 (53.8–104.6)	0.211	0.182	0.018/0.265
	Breast	63.6 ± 24.5 (56.2–71.1)	75.3 ± 23.4 (67.0–80.8)		0.009 *	0.067/0.752
	Prostate	87.5 ± 16.0 (37.1–75.4)	78.6 ± 23.0 (57.3–99.8)		0.651	0.002/0.073
Functional Scales Cognitive functioning	Colorectal	75.0 ± 27.4 (46.3–103.7)	66.7 ± 27.2 (23.4–110.0)	0.660	0.665	0.002/0.072
	Breast	66.3 ± 28.2 (57.7–74.9)	74.1 ± 26.9 (64.5–80.8)		0.374	0.008/0.143
	Prostate	95.8 ± 8.3 (82.6–109.1)	83.3 ± 23.6 (61.5–105.1)		0.915	<0.001/0.051
Functional Scales Social functioning	Colorectal	80.6 ± 22.2 (57.3–103.8)	66.7± 20.4 (34.2–99.2)	0.114	0.184	0.018/0.263
	Breast	82.6 ± 25.2 (74.9–90.2)	82.4 ± 28.3 (72.6–90.5)		0.635	0.002/0.076
	Prostate	104.2 ± 8.3 (90.9–117.4)	85.7 ± 41.3 (47.5–123.9)		0.006 *	0.006/0.125
Symptom Scales/Items Fatigue	Colorectal	35.2 ± 37.5 (−4.1–74.5)	38.9 ± 28.0 (−5.6–83.4)	0.555	0.577	0.003/0.086
	Breast	46.5 ± 22.0 (39.8–53.2)	39.6 ± 28.1 (30.6–48.6)		0.449	0.006/0.117
	Prostate	11.1 ± 15.7 (−13.9–36.1)	23.8 ± 32.4 (−6.1–53.7)		0.972	<0.001/0.050
Symptom Scales/Items Nausea and vomiting	Colorectal	2.8 ± 6.8 (−4.4–9.9)	0.0 ± 0.0 (0.0–0.0)	0.386	0.229	0.015/0.224
	Breast	8.7 ± 13.2 (4.7–12.7)	8.5 ± 16.8 (3.1–13.9)		0.637	0.002/0.076
	Prostate	0.0 ± 0.0 (0.0–0.0)	0.0 ± 0.0 (0.0–0.0)		0.840	<0.001/0.055
Symptom Scales/Items Pain	Colorectal	36.1 ± 35.6 (−1.3–73.5)	37.5 ± 34.4 (−17.2–92.2)	0.410	0.325	0.010/0.165
	Breast	54.2 ± 26.9 (46.0–62.4)	49.1 ± 27.8 (40.2–58.0)		0.619	0.003/0.078
	Prostate	20.8 ± 16.0 (−4.6–46.2)	21.4 ± 31.5 (−7.7–50.6)		0.979	<0.001/0.50
Symptom Scales/Items Dyspnea	Colorectal	16.7 ± 18.3 (−2.5–35.8)	8.3 ± 16.7 (−18.2–34.9)	0.296	0.137	0.022/0.318
	Breast	31.1 ± 30.8 (21.7–40.4)	26.2 ± 30.9 (16.3–36.1)		0.502	0.005/0.102
	Prostate	0.0 ± 0.0 (0.0–0.0)	9.5 ± 16.3 (−5.5–24.6)		0.820	0.001/0.056
Symptom Scales/Items Insomnia	Colorectal	22.2 ± 34.4 (−13.9–58.4)	33.3 ± 27.2 (−10.0–76.6)	0.555	0.947	<0.001/0.051
	Breast	56.8 ± 34.2 (46.4–67.2)	48.3 ± 33.1 (37.7–58.9)		0.030 *	0.047/0.588
	Prostate	8.3 ± 16.7 (−18.2–34.9)	9.5 ± 16.3 (−5.5–24.6)		0.968	<0.001/0.050
Symptom Scales/Items Appetite loss	Colorectal	33.3 ± 42.2 (−10.9–77.6)	16.7 ± 19.3 (−14.0–47.3)	0.407	0.389	0.008/0137
	Breast	21.7± 28.1 (12.7–29.7)	16.2 ± 29.1 (6.9–25.5)		0.372	0.008/0.144
	Prostate	0.0 ± 0.0 (0.0–0.0)	4.8 ± 12.6 (−6.9–16.4)		0.979	<0.001/0.050
Symptom Scales/Items Constipation	Colorectal	16.7 ± 18.3 (−2.5–35.8)	8.3 ± 16.7 (−18.2–34.9)	0.210	0.072	0.033/0.436
	Breast	20.5 ± 27.1 (12.2–28.7)	17.9 ± 22.5 (10.7–25.1)		0.932	<0.001/0.051
	Prostate	8.3 ± 16.7 (−18.2–34.9)	9.5 ± 16.3 (−5.5–24.6)		0.961	<0.001/0.050
Symptom Scales/Items Diarrhea	Colorectal	5.6 ± 13.6 (−8.7–19.8)	0.0 ± 0.0 (0.0–0.0)	0.676	0.276	0.012/0.192
	Breast	9.9 ± 19.8 (3.8–15.9)	8.5 ± 18.1 (2.8–14.3)		0.925	<0.001/0.051
	Prostate	0.0 ± 0.0 (0.0–0.0)	9.5 ± 16.3 (−5.5–24.6)		0.590	0.003/0.083
Symptom Scales/Items Financial difficulties	Colorectal	22.2 ± 40.4 (−20.1–64.6)	8.3 ± 16.7 (−18.2–34.9)	0.888	1.000	<0.001/0.050
	Breast	31.1 ± 34.8 (20.5–41.6)	30.5 ± 33.4 (20.4–41.1)		0.586	0.003/0.084
	Prostate	0.0 ± 0.0 (0.0–0.0)	9.01 ± 21.6 (−10.0–38.5)		1.000	<0.001/0.050

* = significant difference between groups in post-intervention (*p* < 0.05); M = mean ± SD = standard deviation; CI = confidence intervals; ηp^2^ = Partial eta squared. effect size: explains what percentage of the explained variability can be specifically attributed to the effect of the treatment; 1 − *β =* statistical power: is the probability that a study will detect a real effect when that effect actually exists.

**Table A5:** Results of the FACIT-F fatigue questionnaire between groups according to each stage of the disease after implementation of the exercise program in cancer patients.

Variable	Type of Cancer	Control (M ± SD, 95% CI)	Intervention (M ± SD, 95% CI)	*p*-Value Group	*p*-Value	ηp^2^ 1−β
FACIT-F	Colorectal	32.6 ± 17.1 (17.9–53.5)	32.3 ± 10.9 (9.6–49.4)	0.050	0.118	0.024/0.346
	Breast	29.6 ± 9.8 (26.6–32.5)	34.9 ± 10.1 (31.5–38.1)		<0.001 *	0.117/0.956
	Prostate	45.3 ± 6.6 (34.8–55.8)	41.1 ± 13.7 (28.5–53.8)		0.937	<0.001/0.051

FACIT-F = Functional Assessment of Chronic Illness Therapy-Fatigue; * = significant difference between groups in post-intervention (*p* < 0.05); M = mean ± SD = standard deviation; CI = confidence intervals; ηp^2^ = Partial eta squared. effect size: explains what percentage of the explained variability can be specifically attributed to the effect of the treatment; 1 − *β =* statistical power: is the probability that a study will detect a real effect when that effect actually exists.

**Table A6:** Results of functional capacity variables between the control and intervention groups according to type of cancer after implementation of the exercise program in cancer patients.

Variable	Type of Cancer	Control (M ± SD, 95% CI)	Intervention (M ± SD, 95% CI)	*p*-Value Group	*p*-Value	ηp^2^ 1−β
6MWT (meters)	Colorectal	481.4 ± 171.5 (399.5–660.5)	497.5 ± 107.4 (354.2–625.7)	0.020 *	0.208	0.015/0.241
	Breast	492.7 ± 75.4 (469.7–515.6)	538.5 ± 74.9 (515.4–563.2)		0.006 *	0.070/0.790
	Prostate	555.0 ± 41.1 (489.0–621.0)	585.9 ± 75.2 (516.4–655.5)		0.246	0.013/0.211
30s-STST (repetitions)	Colorectal	11.8 ± 4.8 (6.6–17.6)	17.0 ± 2.5 (13.6–22.0)	0.207	0.405	0.007/0.132
	Breast	13.4 ± 4.1 (12.1–14.6)	19.2 ± 16.1 (14.2–24.8)		0.024 *	0.048/0.619
	Prostate	15.1 ± 8.0 (2.4–27.9)	15.6 ± 4.5 (11.4–61.9)		0.828	<0.001/0.055
HGT (kilograms)	Colorectal	28.2 ± 8.9 (18.1–38.6)	24.7 ± 5.9 (17.3–25.4)	0.018 *	0.695	0.001/0.068
	Breast	18.6 ± 5.3 (17.0–20.3)	21.9 ± 6.6 (19.6–23.9)		0.023 *	0.049/0.630
	Prostate	36.8 ± 1.5 (34.5–39.1)	41.2 ± 22.4 † (20.5–61.9)		0.028 *	0.046/0.599

6MWT = six-minute walk test; 30s-STST= 30 s sit-to-stand test; HGT= handgrip test; * = significant difference between groups in post-intervention (*p* < 0.05); † = significant difference between prostate cancer and breast and colon cancer; M = mean ± SD = standard deviation; CI = confidence intervals; ηp^2^ = Partial eta squared. effect size: explains what percentage of the explained variability can be specifically attributed to the effect of the treatment; 1 − *β =* statistical power: is the probability that a study will detect a real effect when that effect actually exists.
